# Modulating Electromagnetic Genes Through Bi-Phase High-Entropy Engineering Toward Temperature-Stable Ultra-Broadband Megahertz Electromagnetic Wave Absorption

**DOI:** 10.1007/s40820-024-01638-4

**Published:** 2025-02-25

**Authors:** Xiaoji Liu, Yuping Duan, Nan Wu, Guangming Li, Yuan Guo, Jiangyong Liu, Ning Zhu, Qiang Wang, Lin Wang, Zichen Xu, Hao Wei, Guojun Wang, Zhijia Zhang, Songsong Zhang, Wenjun Zhou, Teng Ma, Tongmin Wang

**Affiliations:** 1 Qingdao Innovation and Development Base of Harbin Engineering University, Harbin Engineering University, Qingdao, 266000 People’s Republic of China; 2 Key Laboratory of Solidification Control and Digital Preparation Technology, School of Materials Science and Engineering, Dalian University of Technology, Dalian, 116085 People’s Republic of China; 3 National Key Laboratory of Electromagnetic Effect and Security On Marine Equipment, China Ship Development and Design Center, Wuhan, 430205 People’s Republic of China; 4 Wuhan Second Ship Design and Research Institute, Wuhan, 430205 People’s Republic of China

**Keywords:** Bi-phase high-entropy composites, Electromagnetic genes, Electromagnetic wave absorption, Continuous natural resonance, Ultra-broadband

## Abstract

Magnetic absorbers with high permeability have significant advantages in low-frequency and broadband electromagnetic wave (EMW) absorption. However, the insufficient magnetic loss and inherent high conductivity of existing magnetic absorbers limit the further expansion of EMW absorption bandwidth. Herein, the spinel (FeCoNiCrCu)_3_O_4_ high-entropy oxides (HEO) are successfully constructed on the surface of FeCoNiCr_0.4_Cu_0.2_ high-entropy alloys (HEA) through low-temperature oxygen bath treatment. On the one hand, HEO and HEA have different magnetocrystalline anisotropies, which is conducive to achieving continuous natural resonance to improve magnetic loss. On the other hand, HEO with low conductivity can serve as an impedance matching layer, achieving magneto-electric co-modulation. When the thickness is 5 mm, the minimum reflection loss (RL) value and absorption bandwidth (RL <  − 5 dB) of bi-phase high-entropy composites (BPHEC) can reach − 12.8 dB and 633 MHz, respectively. The RCS reduction value of multilayer sample with impedance gradient characteristic can reach 18.34 dB m^2^. In addition, the BPHEC also exhibits temperature-stable EMW absorption performance, high Curie temperature, and oxidation resistance. The absorption bandwidth maintains between 593 and 691 MHz from − 50 to 150 °C. This work offers a new and tunable strategy toward modulating the electromagnetic genes for temperature-stable ultra-broadband megahertz EMW absorption.

**Supplementary Information:**

The online version contains supplementary material available at 10.1007/s40820-024-01638-4.

## Highlights

- The bi-phase FeCoNiCr_0.4_Cu_0.2_/(FeCoNiCrCu)_3_O_4_ high-entropy composites are innovatively constructed for the first time by low-temperature oxygen bath strategy.
- The bi-phase high-entropy composites (BPHEC) can precisely regulate the electromagnetic genes, realizing the ideal ultra-broadband and temperature-stable electromagnetic wave absorption.
- Simultaneously, the formation mechanism of BPHEC during low-temperature oxygen bath and the regulation mechanism of electromagnetic genes are elucidated.

**Supplementary Information:**

The online version contains supplementary material available at 10.1007/s40820-024-01638-4.

## Graphical abstract

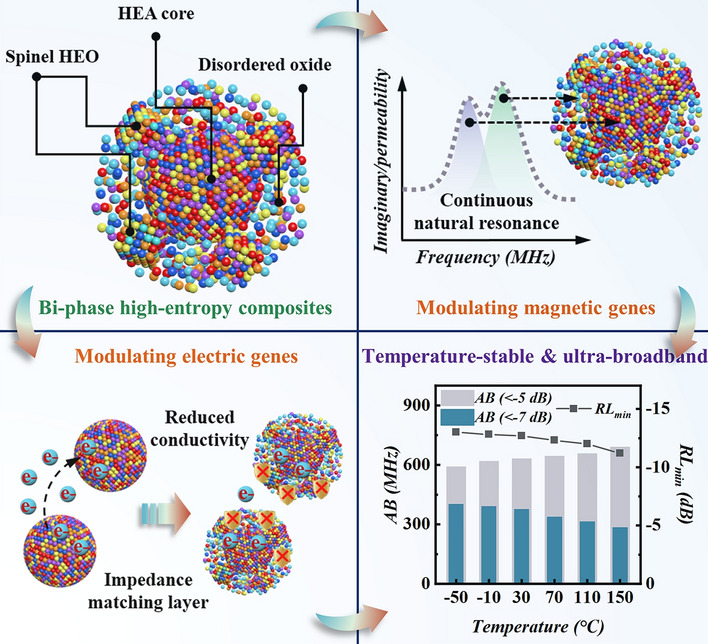

## Introduction

Low-frequency electromagnetic wave (EMW) absorption materials have wide applications in both civil and military fields, and it is of great significance to strengthen the research of EMW absorption materials in the megahertz (MHz) frequency band [1–5]. As the frequency decreases, the coupling degree between wave-impedance and EMW dissipating ability significantly increases, which puts higher requirements on the electromagnetic parameters of EMW absorption materials [2]. The previous studies have shown that magnetic absorbers with high permeability exhibit strong advantages in low-frequency and broadband EMW absorption [6, 7]. In addition, the complex and variable application environments such as polar region, ocean, and desert put forward higher requirements for the environmental adaptability of EMW absorption materials [8–12]. High-entropy alloys (HEA) are considered to be suitable candidates for low-frequency broadband EMW absorption materials with environmental adaptability due to their high designable freedom in composition, microstructure, and morphology [13, 14]. The flake-shaped FeCoNi-based HEA absorbers prepared by a simple mechanical alloying method exhibit high permeability and strong low-frequency EMW absorption performance [15–17]. Meanwhile, due to the four effects of “high-entropy effect,” “cocktail effect,” “severe lattice distortion effect,” and “sluggish diffusion effect,” the FeCoNi-based HEA absorbers exhibit temperature-stable crystalline structure, high Curie temperature, excellent oxidation resistance, and corrosion resistance [9, 18–20]. However, on the one hand, the insufficient magnetic loss of existing HEA absorbers limits the realization of ideal MHz broadband EMW absorption. On the other hand, impedance mismatch due to the inherent high conductivity of metals can also limit the improvement of EMW absorption performance.

As is well known, in a dynamic magnetic field, the complex permeability is composed of the real and imaginary parts (*μ*_*r*_ = *μ*′ − *jμ*″) due to the hysteresis of the magnetization compared to the external magnetic field. The real permeability *μ*′ represents the ability to store magnetic energy. The imaginary permeability *μ*″ represents the ability to consume magnetic field energy, which usually consists of magnetic hysteresis, eddy current, magnetic resonance, and so on. In the previous work, our group prepares FeCoNiCr_0.4_Cu_0.2_ HEA powders with large aspect ratios to break the Snoek’s limit by modulating composition and optimizing preparation process [21, 22]. The natural resonance frequency is modulated by in situ construction of spinel ferrimagnets in HEA [18]. These strategies have been effective in the enhancement and modulation of permeability, which broadens the EMW absorption bandwidth and reduces the thickness of absorbers. The natural resonance, as the main component of magnetic loss in the microwave band, has been commonly modulated to achieve the enhancement of the imaginary permeability by regulating its peak value and peak position. Nevertheless, there is a lack of attention to the shape and width of the natural resonance peak (“magnetic genes”). Typically, single-phase magnetic materials have only one natural resonance peak. This means that the imaginary permeability increases monotonically with increasing frequency before reaching the peak and decreases monotonically with increasing frequency after the resonance peak. This single natural resonance peak is obviously not conducive to enhancing the magnetic loss capability of the absorber and realizing broadband EMW absorption. The ideal state is multiple or continuous peaks to broaden the natural resonance peak width. The construction of the multiphase structure offers the possibility of broadening the natural resonance peak.

Recently, some advantages of multiphase materials in the field of EMW absorption have been discovered [23–26]. For example, a unique core–shell bi-magnetic BaFe_(12−*x*)_Co_*x*_O_19_@Fe_3_O_4_ microsphere exhibits robust exchange coupling interaction and competitive EMW absorption performance in the lower­frequency range [2]. Fluorine ion (F^−^) regulation engineering shows that the NiCo_2_S_4_/Co_1−*x*_S/Co(OH)F composite prepared by F^−^-induced new phase formation strategy exhibits superior EMW absorption performance in the low-frequency range compared to F^−^ bath and F^−^ doping strategies [27]. An in situ conductive heterogeneous phase is constructed in the (Fe_0.2*x*_Co_0.2_Ni_0.2_Cr_0.2_Mn_0.2_)_3_O_4_ (*x* = 1–5) high-entropy oxides (HEO) via a treatment in reductive circumstance to form a dual-phase composite [28]. The dual-phase composite can regulate impedance matching and loss capability by modulating the conductive phase, ultimately realizing the enhancement of EMW absorption performance. Therefore, in situ construction of bi-phase high-entropy composite (BPHEC) is feasible for realizing MHz broadband EMW absorption [29]. On the one hand, HEO and HEA have different natural resonance frequencies, which are expected to construct multiple natural resonance peaks, thereby improving magnetic losses in the MHz frequency band. On the other hand, the conductivity of HEO is lower than that of HEA, and impedance matching can be controlled by changing the content of HEO. However, the construction of bi-phase materials often destroys the original composition, microstructure, and morphology of the absorbers, which is not conducive to the precise regulation of the electromagnetic properties [30]. It is a challenge to realize precise construction of bi-phase magnetic absorbers while ensuring the advantages of the original unique microstructure and morphology.

In this study, we innovatively construct bi-phase FeCoNiCr_0.4_Cu_0.2_/(FeCoNiCrCu)_3_O_4_ high-entropy composites by low-temperature oxygen bath strategy. The BPHEC can precisely regulate the electromagnetic genes while maintaining the original crystalline structure and morphology. This is beneficial for achieving ideal ultra wideband and temperature-stable electromagnetic wave absorption. Simultaneously, we elucidate the formation mechanism of BPHEC during low-temperature oxygen bath and the regulation mechanism of electromagnetic genes. Inspired by this, EMW absorption coatings with impedance gradient characteristics are designed, and the unique advantages of impedance gradient structure in the field of EMW stealth are verified through the computational simulation. This provides a new design idea to construct bi-phase composites in situ and opens a new door for the regulation of electromagnetic genes.

## Experiments

### Raw Materials and Preparation of BPHEC

**Fig. 1:**
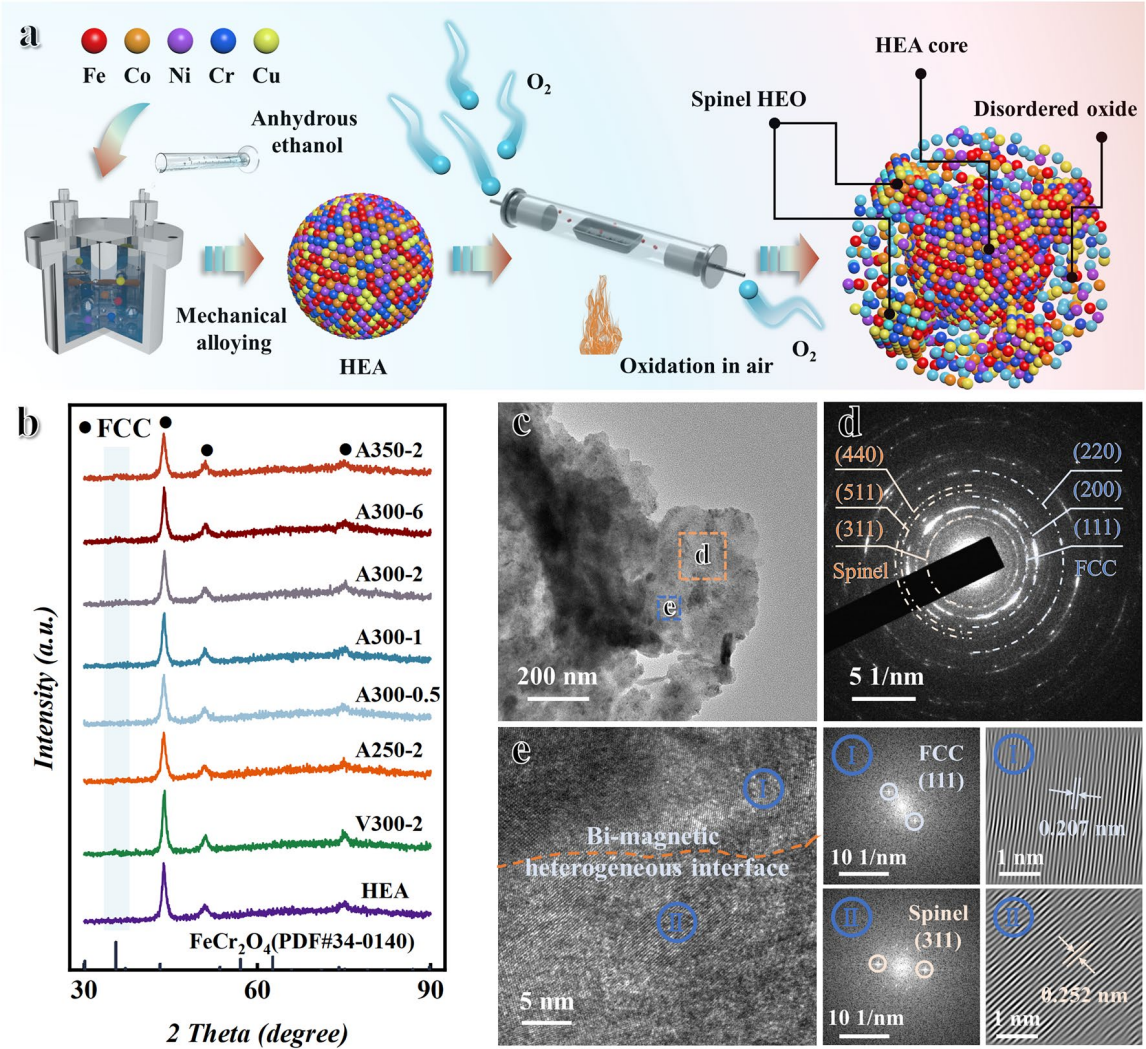
Preparation process and phase structure analysis. **a** Schematic illustration of the preparation for BPHEC. **b** XRD image of HEA, V300-2, and HEA oxidized at different temperatures and times. **c** TEM, **d** SEAD, and **e** HRTEM, FFT, and IFFT images of A300-1

Fe–Co–Ni–Cr–Cu five metal powders (detailed parameters in Supporting Information) are bought from Aladdin Bio-Chem Technology Co., Ltd. (Shanghai, China). The preparation procedure of FeCoNiCr_0.4_Cu_0.2_ (atomic ratio is 1:1:1:0.4:0.2) HEA powders is referred to the previous research [18]. The prepared HEA powders are oxidized in a tube annealing furnace (OTX-1200X) under air atmosphere (1 atm) for the preparation of BPHEC. The oxygen bath temperature and time are set to 2 h at 250 °C, 0.5, 1, 2, and 6 h at 300 °C, and 2 h at 350 °C (designated as A250-2, A300-0.5, A300-1, A300-2, A300-6, and A350-2), respectively. In addition, the HEA powders are annealed under vacuum at 300 °C for 2 h as a comparison sample, named V300-2. The detailed preparation process is shown in Fig. 1a.

### Microstructure and Morphology Characterization

The phase structure of the samples is examined by an X-ray diffraction (XRD-6000) and a field emission transmission electron microscope (TEM, JEM-F200). The scanning electron microscope (SEM, SU5000) with an energy-dispersive spectroscopy (EDS) is used to examine the morphology, elements mapping, and elements content. The surface chemical structure of the samples is characterized by an X-ray photoelectron spectroscopy (XPS, ESCALAB XI+).

### Electromagnetic Performance Testing

The hysteresis loop and thermomagnetic curve are measured with the vibrating sample magnetometer (VSM7404-S) under an applied field of 12,000 Oe. Keysight E4991B impedance analyzer with Espec SU-262 Benchtop temperature chamber is used to test the complex permeability and complex permittivity under variable temperature. The samples are prepared into a concentric ring with inner diameter of 3.1 mm and outer diameter of 8 mm under 4 MPa pressure to investigate the complex permeability. Moreover, the samples are pressed into disk with diameter of 18 mm to measure complex permittivity. The measured electromagnetic parameters are used to calculate the reflection loss. The conductivity is tested by the RTS-9 dual electrical measuring four probe tester. The thermogravimetric curve is tested by the TGA/SDTA851e thermogravimetric analyzer with the heating rate is 10 °C min^−1^ in air atmosphere.

### Computational Simulations

This article simulates the radar cross-section (RCS) of samples using CST Studio Suite 2020 software. The selected frequency is 750 MHz. Based on the widely studied metal back model, the PEC and the coating layer are placed on the X–O–Y plane, and the propagation of the plane wave (PW) is along from the positive to the negative direction of the Z-axis. The 3D size of the PEC and absorber model is set as 1000 × 1000 × 1 and 1000 × 1000 × 5 mm^3^, respectively. The boundary conditions are applied with *x*, *y*, and *z* directions. Open (add space) boundary conditions are set in all directions. Theta and phi are defined as the detection angle and the observation angle, respectively.

## Results and Discussion

### Microstructure, Composition, and Morphology

In the previous work, spinel ferrimagnets are constructed in situ in HEA by annealing treatment, which realizes the modulation of the natural resonance peak position and the improvement of EMW absorption efficiency [18]. However, sintering between the HEA powders due to high-temperature annealing treatment reduces the shape anisotropy and the initial permeability, which is not conducive to broadening the EMW absorption bandwidth [31]. At the same time, the annealing treatment leads to permittivity enhancement and impedance mismatch due to the improvement of crystallinity. Therefore, in this study, a low-temperature oxygen bath strategy is used to introduce oxygen ion on the surface of the HEA, aiming to form a bi-phase HEA/HEO composite material to synergistically modulate the electromagnetic genes and achieve efficient broadband EMW absorption. Figure 1a shows the preparation process of HEA powders and the schematic diagram of oxygen ion introduction. XRD image shows that the five metal elements are fully alloyed and form simple solid solution structure with FCC phase (corresponding to (111), (200), and (220)) after mechanical alloying, as shown in Fig. 1b. The diffraction peaks (peak height and peak width) corresponding to FCC in HEA oxidized at different temperatures and times are basically unchanged, which indicates that the crystallinity of samples has not changed much. As the oxygen bath temperature and time increases, the diffraction peak (311) corresponding to spinel FeCr_2_O_4_ phase appears. In order to further analyze the crystal structure of the samples after the oxygen bath treatment, the microstructure of the A300-1 sample is characterized by TEM. TEM bright-field image (Fig. 1c) shows that HEA powders exhibit a flake-shaped structure. The SEAD shows that there are diffraction rings corresponding to the two phase structures (FCC phase and spinel phase) in A300-1 sample, as shown in Fig. 1d. Meanwhile, as shown in Fig. 1e, the HRTEM further demonstrates the existence of bi-phase structures of FCC and spinel in the A300-1 sample, and the existence of a clear interface between the two phases (shown by the yellow line in Fig. 1e). We perform the FFT and IFFT for the two phases separated by the interface, respectively. The lattice fringes of 0.252 and 0.207 nm correspond to (311) crystal plane of spinel phase and (111) crystal plane of FCC phase, respectively. This indicates the formation of bi-phase FCC/spinel composites after oxygen bath treatment. However, the diffraction peak corresponding to spinel phase is not evident in the XRD plot. This is because oxygen ion presents only on the surface of the HEA and the low content of the spinel phase relative to the whole.

**Fig. 2:**
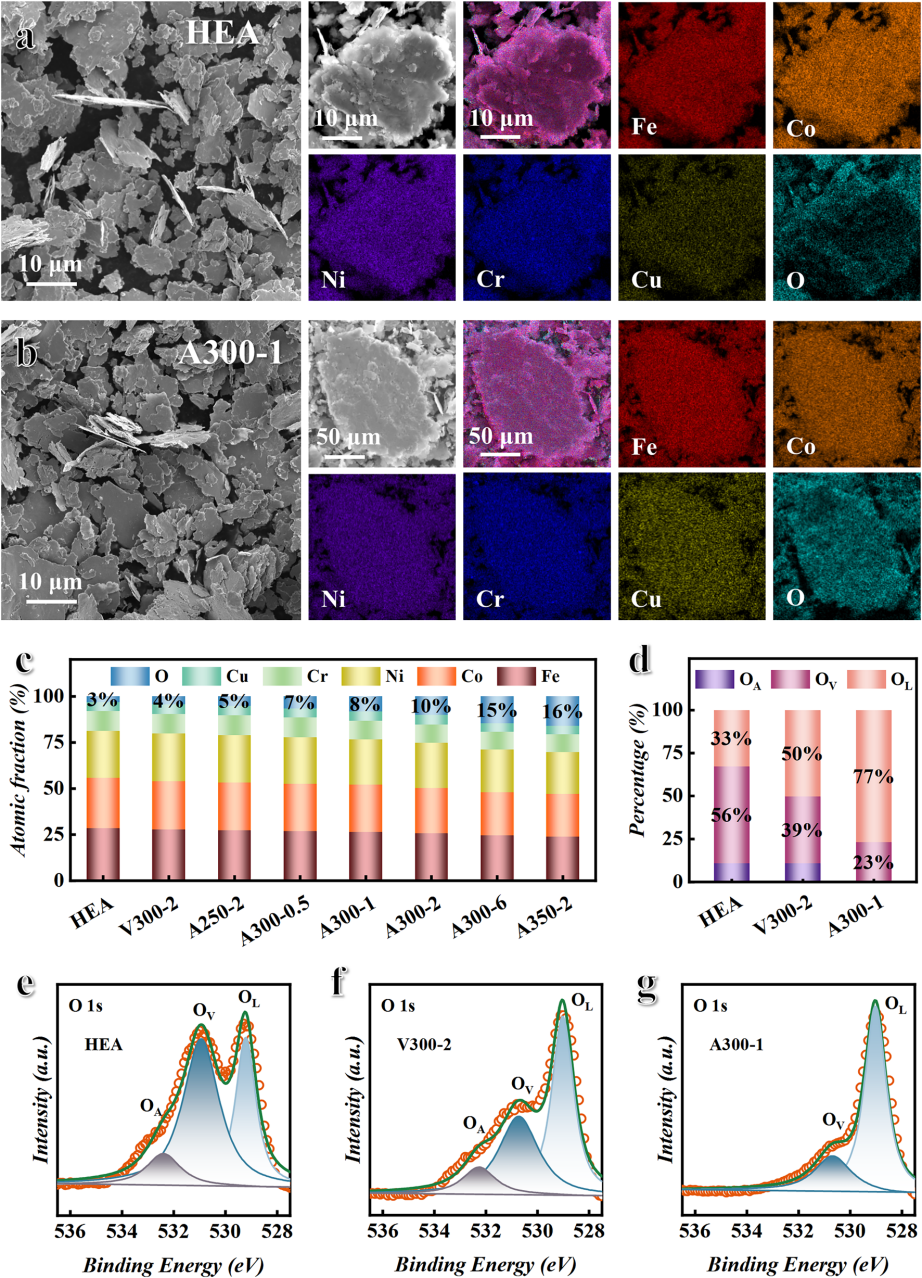
Analysis of the morphology, composition, and surface chemical state. SEM images and elements mappings of **a** HEA and** b** A300-1. **c** Surface element atomic fraction of HEA, V300-2, and HEA oxidized at different temperatures and times. **d** Oxygen percentage of O_L_, O_V_, and O_A_ for HEA, V300-2, and A300-1. O 1s XPS spectra of **e** HEA, **f** V300-2, and **g** A300-1

In order to further investigate the content and type of introduced oxygen elements, the composition and surface chemical state of samples are characterized by SEM and XPS. As shown in Fig. 2a, the HEA powders prepared by mechanical alloying have a uniform elemental distribution, and a small amount of oxygen element are introduced during the preparation process due to the addition of anhydrous ethanol. Figure 2b shows that the Fe–Co–Ni–Cr–Cu five metal elements remain uniformly distributed after oxygen bath at 300 °C for 1 h, and the content of oxygen elements increases significantly. To further quantify the oxygen elemental content, we analyze the surface composition of selected areas for HEA oxidized at different temperatures and times, as shown in Figs. S1 and S2. The statistical results are shown in Fig. 2c, the content of oxygen elements on the surface of the samples gradually increases, and the content of five metal elements decreases proportionally with the increase in the oxygen bath temperature and time. Meanwhile, XPS is used to analyze the chemical states of Fe, Co, Ni, Cr, Cu, and O elements on the surface of the samples. Figure S3 presents the full XPS spectrum, and seven typical peaks are observed, corresponding to C 1*s*, O 1s, Cr 2*p*, Fe 2*p*, Co 2*p*, Ni 2*p,* and Cu 2*p*, respectively. In the O 1s spectrum, the peaks at 529.2, 530.9, and 532.4 eV are assigned to lattice oxygen (O_L_), oxygen vacancy (O_V_), and surface absorbed oxygen (O_A_), respectively [32]. Figure 2e shows that there are three types of oxygen elements in HEA powders without oxygen bath treatment. After vacuum annealing treatment at 300 °C for 2 h, the O_V_ content of the V300-2 sample decreases, as shown in Fig. 2f. Because the oxygen bath process provides sufficient diffusion activation energy to reduce internal stress and defects of the samples. After oxygen bath at 300 °C for 1 h, the proportion of O_L_ increases, and the O_A_ basically disappears, as shown in Fig. 2g. The statistical results indicate that the proportion of O_L_ in A300-1 sample reaches 77%, as shown in Fig. 2d. The XPS spectra of Cr 2*p*, Fe 2*p*, Co 2*p*, Ni 2*p,* and Cu 2*p* are subjected to peak splitting processing. As shown in Fig. S4, the peak area corresponding to the Fe^3+^ (712.7 and 725.5 eV belong to Fe^3+^) on the surface of the sample after oxygen bath treatment increases, while the area corresponding to the Fe^2+^ (710.2 and 723.5 eV belong to Fe^2+^) decreases. The peak area corresponding to the Co^3+^ (779.6 and 794.8 eV belong to Co^3+^) of the sample after oxygen bath treatment increases, while the area corresponding to the Co^2+^ (785.1 and 802.4 eV belong to Co^2+^) decreases. The peak area corresponding to the Ni^2+^ (860.6 and 879.4 eV belong to Ni^2+^) of the sample after oxygen bath treatment increases. The peak corresponding to the Cu^0^ (931.3 and 951.6 eV belong to Cu^0^) of the sample after oxygen bath treatment disappears, while the peak corresponding to the Cu^2+^ (933.3 and 954.1 eV belong to Cu^2+^) appears. The Cr element maintains Cr^3+^ state after oxygen bath treatment due to its strong affinity with oxygen element. In conclusion, the metal elements on the surface of HEA are oxidized to high valence metal ions in the oxygen bath process, and the introduced oxygen elements are transformed from O_A_ and Ov to O_L_. The spinel (FeCoNiCrCu)_3_O_4_ HEO are fabricated on the surface of HEA by oxygen bath treatment. In addition, Fig. 2a, b indicates that the sample after oxygen bath at 300 °C for 1 h still maintains its original advantage of large aspect ratio. However, as shown in Fig. S5, the A350-2 sample exhibits slight sintering phenomenon, which will reduce shape anisotropy and initial permeability.

### Electromagnetic Performance

**Fig. 3:**
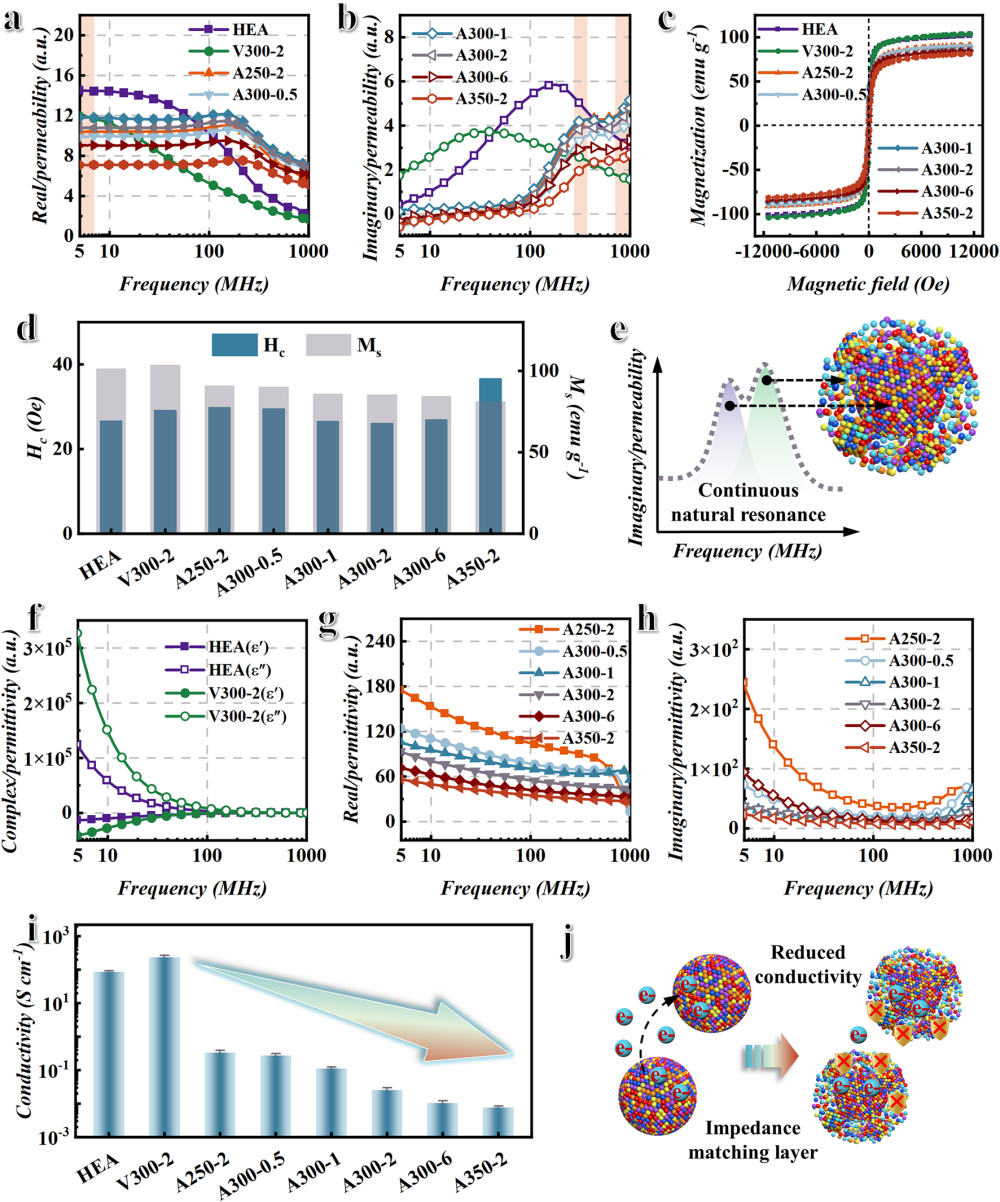
Analysis of elecromagnetic performance. **a** Real permeability *μ*′ and **b** imaginary permeability *μ*″ of HEA, V300-2, and HEA oxidized at different temperatures and times versus frequency. **c** Hysteresis loop, **d** saturation magnetization M_s_, and coercivity H_c_ of HEA, V300-2, and HEA oxidized at different temperatures and times in an applied magnetic field range of − 12,000–12,000 Oe. **e** Schematic diagram of BPHEC to modulate magnetic genes. **f** Real permittivity ε′ and imaginary permittivity *ε*″ of HEA, V300-2 versus frequency. **g** Real permittivity *ε*′ and **h** imaginary permittivity *ε*″ of HEA oxidized at different temperatures and times versus frequency. **i** Conductivity of HEA, V300-2, and HEA oxidized at different temperatures and times. **j** Schematic diagram of BPHEC to modulate electric genes

Figure 3a, b exhibits the real permeability *μ*′ and imaginary permeability *μ*″ of HEA, V300-2, and HEA oxidized at different temperatures and times versus frequency, respectively. The yellow shaded area in Fig. 3a represents the initial permeability (before the cut-off frequency) of samples. As shown in Fig. 3a, the initial permeability of samples after annealing and oxygen bath treatment decreases. And the initial permeability of the samples increases and then decreases with the increase in oxygen bath temperature and time. The hysteresis loop of samples is measured to analyze the variation of permeability, as shown in Fig. 3c. The statistical results of saturation magnetization and coercivity of samples are shown in Fig. 3d and Table S2. Figure 3d shows that the saturation magnetization and coercivity of samples have increased after vacuum annealing, which is due to the increase in crystallinity and the decrease in internal stress. The saturation magnetization of samples after oxygen bath treatment decreases due to the formation of HEO. With the increase in oxygen bath temperature and time, the coercivity of samples decreases first and then increases. After the oxygen bath treatment, the samples still maintain a small coercivity and good soft magnetic properties, which indicates that the proportion of HEO constructed by the oxygen bath treatment is relatively small. However, the coercivity of A350-2 sample increases sharply. This may be because the sintering caused by oxygen bath at 350 °C for 2 h reduces shape anisotropy. In a word, the initial permeability of samples basically meets the rule that it is proportional to the saturation magnetization and inversely proportional to the coercivity. The A300-1 sample has high initial permeability due to its low coercivity and high saturation magnetization. The yellow shaded area in Fig. 3b represents the position of the natural resonance peak. As shown in Fig. 3b, the vacuum annealed sample V300-2 has only a single natural resonance peak, while all the oxygen bath treated samples have two natural resonance peaks. And the samples treated with oxygen bath exhibit a high imaginary permeability in the frequency range of 300–1000 MHz. This indicates that the construction of the BPHEC successfully realizes the continuous natural resonance peak and the improvement of magnetic loss. Figure 3e shows the mechanism diagram of realizing continuous natural resonance peak for the BPHEC. Due to the large magnetocrystalline anisotropy of spinel HEO, the corresponding natural resonance peak frequency is high [33–35]. The magnetocrystalline anisotropy of FCC HEA is small, and the corresponding natural resonance peak frequency is low. In addition, the exchange coupling between the bi-magnetic heterogeneous interfaces makes the sample finally form a continuous natural resonance peak. Figure 3f shows real permittivity and imaginary permittivity of HEA powders and V300-2 sample. Due to the inherent high conductivity of metals, the real permittivity of HEA is negative, and the imaginary permittivity is more than 100,000, which is obviously not conducive to impedance matching and efficient EMW absorption [36]. Compared with HEA, V300-2 sample has higher complex permittivity (Fig. 3f) and conductivity (Fig. 3i) due to the increase in crystallinity caused by annealing. The construction of HEO significantly reduces the complex permittivity (Fig. 3g, h) and conductivity (Fig. 3i). Moreover, with the oxygen bath temperature and time increasing, the complex permittivity and conductivity of BPHEC show a decreasing trend. Figure 3j shows the mechanism of BPHEC regulating electric genes. Due to the low conductivity, the HEO acts as an insulating layer, reducing the transmission efficiency of free electrons and avoiding the overlapping of HEA powders with high conductivity to form a conductive network. This is equivalent to building an impedance matching layer on the surface of HEA powders, which can make more EMW incident into the absorbers [37]. Therefore, the construction of BPHEC can not only regulate magnetic genes to increase magnetic loss, but also regulate electric genes to improve impedance matching.

### EMW Absorption Performance

**Fig. 4:**
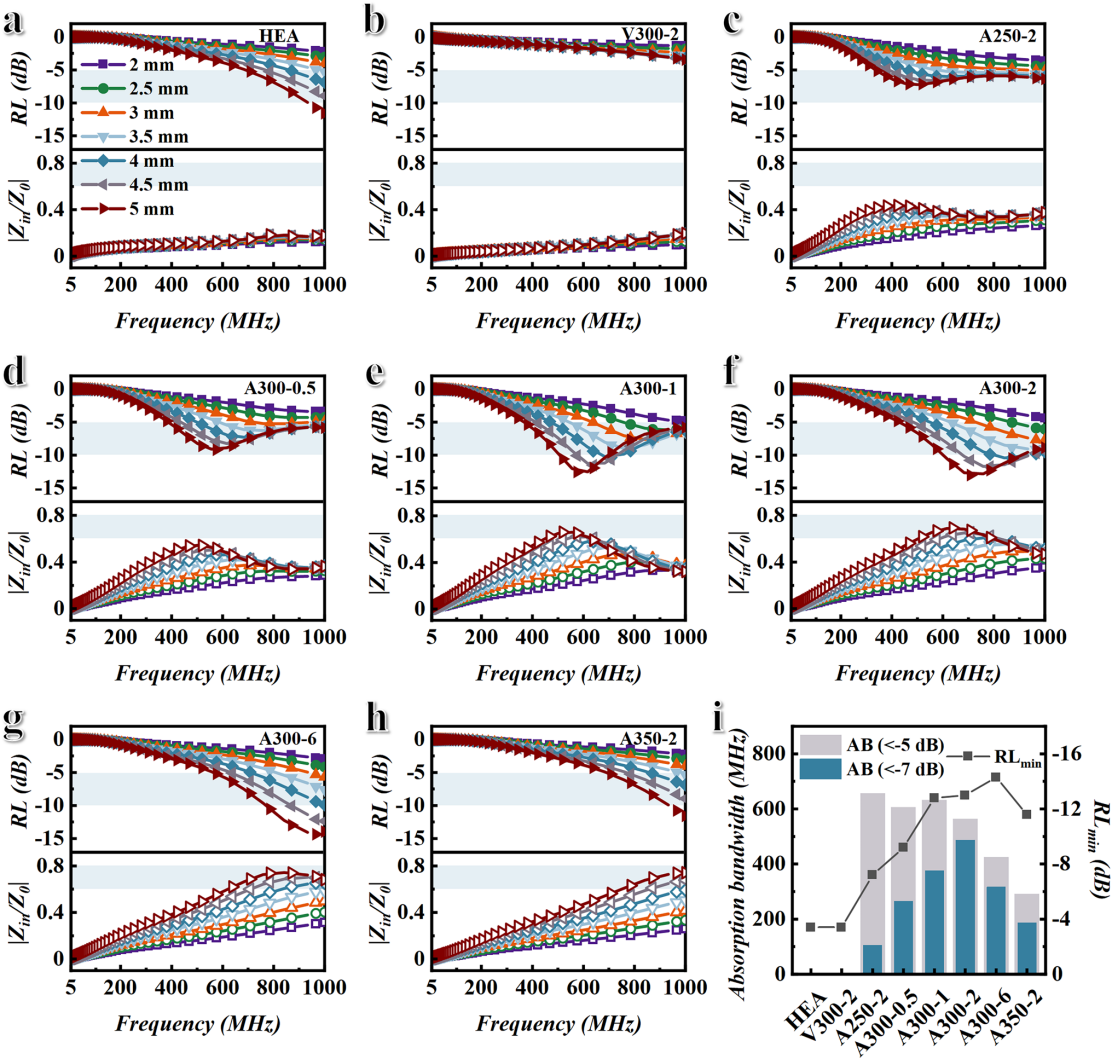
Analysis of EMW absorption performance. **a–h** Reflection loss and impedance matching diagrams of HEA, V300-2, and HEA oxidized at different temperatures and times. **i** Absorption bandwidth AB and minimum reflection loss value RL_min_ of HEA, V300-2, and HEA oxidized at different temperatures and times versus thickness

The reflection loss (RL) of HEA, V300-2, and BPHEC is calculated by the transmission line theory, described as follows [38–42]:

1 $${\text{RL}}\left( {{\text{dB}}} \right) = 20\log_{10} \left| {\left( {Z_{{{\text{in}}}} - Z_{0} } \right)/\left( {Z_{{{\text{in}}}} + Z_{0} } \right)} \right|$$

2 $$\frac{{Z_{{{\text{in}}}} }}{{Z_{0} }} = \sqrt {\frac{{\mu_{r} }}{{\varepsilon_{r} }}} \tanh \left( {j\frac{2\pi tf}{c}\sqrt {\mu_{r} \varepsilon_{r} } } \right)$$

where *Ζ*_in_ represents the input impedance, *Ζ*_0_ represents the characteristic impedance of the transmission line, *t* means thickness of absorbers, and *c* represents the speed of light in free space. Figure 4a–h shows the RL and impedance matching of HEA, V300-2, and BPHEC at different thicknesses. As shown in Fig. 4a–h, the HEA powders after oxygen bath treatment have more efficient EMW absorption performance. This is attributed to higher magnetic loss and better impedance matching (the closer |*Z*_in_/*Z*_0_| is to 1, the better the impedance matching performance) of BPHEC [43]. The statistical results of absorption bandwidth and minimum reflection loss (RL_min_) of HEA, V300-2, and BPHEC with a thickness of 5 mm are shown in Fig. 4i. Figure 4i shows that A300-1, A300-2, and A300-6 samples exhibit high RL. The RL_min_ of A300-1, A300-2, and A300-6 samples can reach − 12.8, − 13, and − 14.3 dB, respectively. All three samples can achieve over 90% EMW absorption value. This is attributed to excellent impedance matching and attenuation coefficient (Fig. S6) of BPHEC. At the same time, Fig. 4i shows that the HEA powders after oxygen bath treatment exhibit ultra-broadband EMW absorption. When the thickness is 5 mm, the absorption bandwidth of A300-1 sample can reach 633 MHz (RL <  − 5 dB, absorption value more than 68.4%) and 376 MHz (RL <  − 7 dB, absorption value more than 80%), and the absorption bandwidth of A300-2 sample can reach 563 MHz (RL <  − 5 dB) and 487 MHz (RL <  − 7 dB). According to the Planck–Rozanov limit, this is mainly due to the large initial permeability of the samples [44, 45].

### Simulation of BPHEC for Effective EMW Absorption

**Fig. 5:**
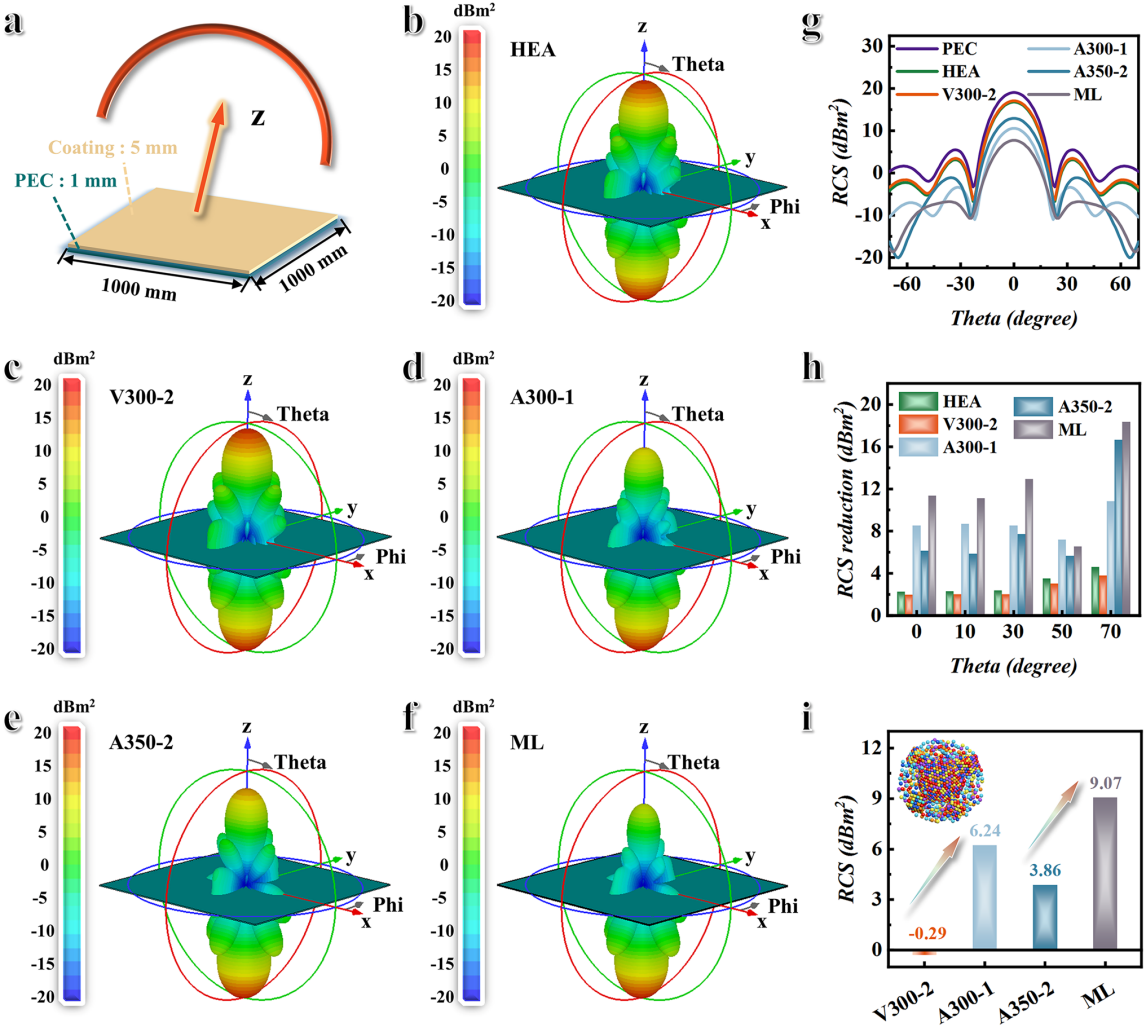
CST simulation results. **a** The model of simulation, rectangle PEC substrate (1000 × 1000 × 1 mm^3^) covered with EMW absorption coating layer (1000 × 1000 × 5 mm^3^). **b–f** Three-dimensional RCS plots of the PEC substrate covered with different EMW absorption materials at 750 MHz. **g** RCS plot in the cartesian coordinate system under certain detecting angles. **h** RCS reduction values of HEA, V300-2, A300-1, A300-2, and ML. **i** Compared with HEA, the RCS reduction values of V300-2, A300-1, A300-2, and ML

Radar cross-section (RCS) is a crucial indicator that reflects the obtained absorbers’ actual far-field condition microwave absorption performance, which can be calculated as follows [46, 47]:

3 $$\sigma\left( {{\text{dBm}}^{2} } \right) = 10\log \left[ {\frac{4\pi S}{{\lambda^{2} }}\left| {\frac{{E_{{\text{s}}} }}{{E_{{\text{i}}} }}} \right|^{2} } \right]$$

where *S*, *λ*, *E*_s_, and *E*_i_ are the area of the simulated plate, wavelength of the incident EMW, and electric field intensities of the transmitting waves and the receiving waves, respectively. Usually, absorbers with excellent EMW absorption performance are coated on the surface of military equipment to reduce the RCS of military targets. To assess the practical application potential of absorbers, CST STUDIO SUITE 2020 software is employed to simulate the RCS of a rectangular perfect electric conductor (PEC) plate substrate (size: 1000 × 1000 × 1 mm^3^) coated with 5 mm thickness of absorbers, respectively. The simulated model is shown in Fig. 5a, plane wave (PW) is served as an excitation source, and the direction of PW is along the negative Z-axis. Theta is defined as the detection angle. HEA, V300-2, A300-1, and A350-2 samples are selected as the coating materials. Meanwhile, inspired by the impedance matching layer of BPHEC, a multilayer EMW absorption coating (consisting of five layers of A300-0.5, A300-1, A300-2, A300-6, and A350-2 from bottom to top, the thickness of each layer is 1 mm) with impedance gradient characteristics is designed, denoted by ML. Figure 5b–f displays the 3D RCS plots for the PEC substrate covered with different absorbers at 750 MHz. Figure 5b–f shows that all absorbers have 3D radar wave reflection signals with similar shape, but with different reflection intensities. According to the color change of the 3D RCS plots, it can be clearly seen that the reflection intensity of A300-1 and ML samples is relatively low. This indicates that A300-1 and ML samples can attenuate more EMW energy to reduce reflected signals. The 2D incident angle-dependent (0°–180°) RCS curves of PEC, HEA, V300-2, A300-1, A350-2, and ML are shown in Fig. 5g. Compared to PEC, the RCS values of A300-1 and ML samples have significantly decreased. To further estimate the intrinsic EMW absorption ability of different absorbers, the RCS reduction value at four certain detection angels (0°, 30°, 60°, and 90°) is obtained by subtracting the RCS value of the PEC layer (Fig. 5h). Figure 5h shows that all EMW absorbers contribute to reducing the RCS of military equipment. The RCS reduction value of A300-1 sample can reach 10.83 dB m^2^. The RCS reduction value of ML sample with impedance gradient characteristic can reach 18.34 dB m^2^. This indicates that the impedance gradient characteristic can not only be used for modulating the electromagnetic performance of materials, but also for macroscopic design of stealth coatings. In addition, the RCS reduction values of each sample relative to HEA (with a detection angel of 0°) are also calculated, as shown in Fig. 5i. Figure 5i shows that the A300-1 sample has an RCS reduction value of 6.24 dB m^2^ compared to the HEA sample due to its excellent magnetic loss and impedance matching. Due to its impedance gradient characteristic, the RCS reduction value of ML sample can reach 9.07 dB m^2^ compared to the HEA sample.

### Variable Temperature EMW Absorption and Oxidation Resistance Performance

**Fig. 6:**
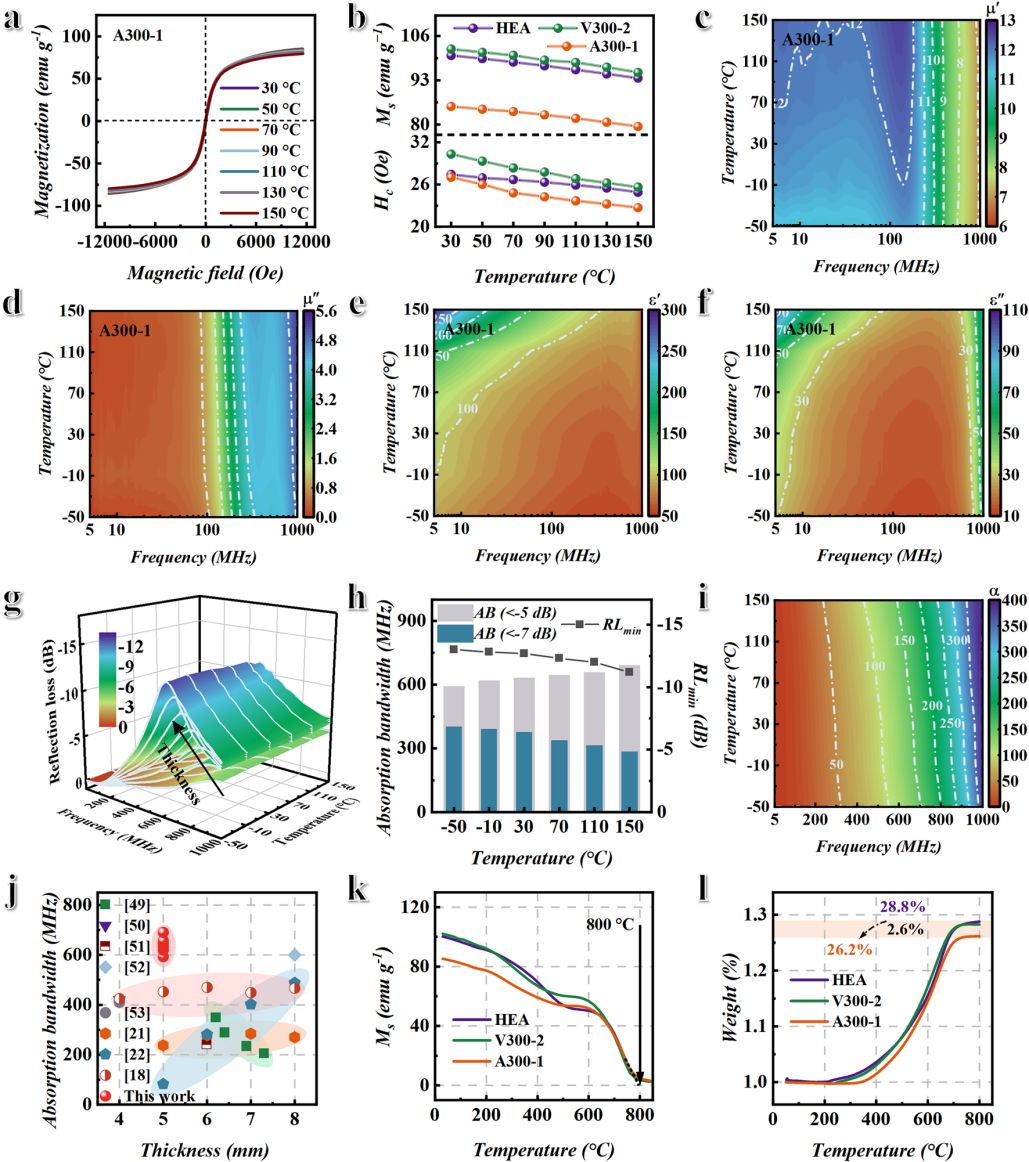
Analysis of variable temperature EMW absorption and oxidation resistance performance. **a** Hysteresis loop, **b** saturation magnetization M_s_, and coercivity H_c_ of HEA, V300-2, and A300-1 versus temperature. **c** Real permeability *μ*′ and **d** imaginary permeability *μ*″ of A300-1 versus temperature. **e** Real permittivity *ε*′ and **f** imaginary permittivity *ε*″ of A300-1 versus temperature. **g** Three-dimensional reflection loss, **h** absorption bandwidth AB and minimum reflection loss value RL_min_ of A300-1 sample with a thickness of 5 mm versus temperature. **i** The attenuation coefficient *α* of A300-1 from − 50 to 150 °C. **j** Comparative chart of absorption bandwidth and thickness for MHz electromagnetic absorbers [18, 21, 22, 49–53]. **k** Thermomagnetic curve and **l** thermogravimetric curve of HEA, V300-2, and A300-1

The practical application environment of radar stealth materials is usually harsh, such as desert, polar region, etc. This not only requires the absorber to have high efficiency and broadband EMW absorption performance, but also requires the absorber to have excellent temperature stability and oxidation resistance. According to the previous studies, temperature-stable permeability is the key to achieve temperature-stable EMW absorption performance, and the temperature dependence of permeability is mainly determined by coercivity and saturation magnetization [48]. Therefore, the hysteresis loops of the HEA, V300-2, and A300-1 samples from − 50 to 150 °C are tested to analyze permeability, as shown in Figs. 6a and S7. The statistical results of saturation magnetization and coercivity of the HEA, V300-2, and A300-1 samples are shown in Fig. 6b. Figure 6b shows that the HEA, V300-2, and A300-1 samples have stable saturation magnetization and a similar trend of change with increasing temperature. However, compared with HEA sample, the coercivity of V300-2 and A300-1 samples decreases faster with the increase in temperature. This may be due to grain growth caused by the annealing process. But it also shows that the temperature dependence of coercivity and saturation magnetization is not significantly affected by the HEO. At the same time, the complex permeability (Fig. 6c, d) and complex permittivity (Fig. 6e, f) of A300-1 sample with excellent EMW absorption performance are tested from − 50 to 150 °C, and calculated the reflection loss (Fig. 6g). Figure 6c, d indicates that the complex permeability of A300-1 sample is relatively stable with increasing temperature, which is similar to the trend of change in HEA sample (Fig. S8). In addition, due to the presence of HEO with low conductivity on the surface of the A300-1 sample, the A300-1 sample also exhibits temperature-stable complex permittivity at − 50–150 °C. Due to the temperature-stable permeability and permittivity, the A300-1 sample exhibits temperature-stable EMW absorption performance at − 50–150 °C, as shown in Fig. 6g. Moreover, Fig. 6g shows that the RL peak of the sample shifts to low frequency as the thickness increases. The statistical results of absorption bandwidth and RL_min_ for A300-1 sample with a thickness of 5 mm are shown in Fig. 6h. Figure 6h shows that the A300-1 sample has temperature-stable absorption bandwidth and RL_min_. This is mainly attributed to the temperature-stable attenuation coefficient (Fig. 6i) and impedance matching (Fig. S10) at − 50–150 °C. The absorption bandwidth (RL <  − 5 dB) is 593–691 MHz from − 50 to 150 °C and increases slightly as temperature increases. This is because the permeability of A300-1 sample slightly increases with increasing temperature. The absorption bandwidth (RL <  − 7 dB) and RL_min_ decrease slightly with the increase in temperature. This is because the permittivity increases as temperature increases, resulting in impedance mismatch. Compared with other MHz electromagnetic absorption materials, the BPHEC can not only obtain broadband MHz electromagnetic absorption performance at a relatively thin thickness, but also have excellent temperature stability, as shown in Fig. 6j and Table S3 [18, 21, 22, 49–53]. The thermomagnetic curve shows that the Curie temperature of the A300-1 sample can reach 800 °C, as shown in Fig. 6k. From the thermogravimetric curve in Fig. 6l, the A300-1 sample has better oxidation resistance compared with the HEA and V300-2 samples. The weight increase is only 26.2% at 800 °C, which is 2.6% lower than the HEA sample. This is mainly because the HEO isolation layer is formed on the surface of HEA. In summary, BPHEC not only regulates electromagnetic genes to achieve efficient broadband EMW absorption, but also exhibits excellent temperature stability and oxidation resistance.

## Conclusions

In summary, the spinel (FeCoNiCrCu)_3_O_4_ high-entropy oxides (HEO) are successfully constructed on the surface of FeCoNiCr_0.4_Cu_0.2_ high-entropy alloys (HEA) through low-temperature oxygen bath treatment. The metal elements on the surface of HEA are oxidized to high valence metal ions in the oxygen bath process, and the introduced oxygen elements are transformed from oxygen vacancy and surface absorbed oxygen to lattice oxygen. When the oxygen bath temperature is 300 °C, the bi-phase high-entropy composites (BPHEC) can achieve regulation of electromagnetic genes while maintaining its original crystal structure and large aspect ratio. On the one hand, HEO and HEA have different magnetocrystalline anisotropies, which is conducive to achieving continuous natural resonance to improve magnetic loss. On the other hand, HEO with low conductivity can serve as an impedance matching layer, achieving magneto-electric co-modulation. When the thickness is 5 mm, the minimum reflection loss value (RL_min_) and absorption bandwidth (RL <  − 5 dB) of BPHEC can reach − 12.8 dB and 633 MHz, respectively. Meanwhile, the RCS reduction value of BPHEC can reach 10.83 dB m^2^. The RCS reduction value of multilayer sample with impedance gradient characteristic can reach 18.34 dBm^2^. This indicates that the impedance gradient characteristic can not only be used for modulating the electromagnetic performance of materials, but also for macroscopic design of stealth coatings. In addition, BPHEC also exhibits temperature-stable electromagnetic wave absorption performance, high Curie temperature, and oxidation resistance. The absorption bandwidth maintains between 593 and 691 MHz from − 50 to 150 °C. The Curie temperature can reach 800 °C, and the weight increase is only 26.2% at 800 °C. This indicates that BPHEC not only achieves temperature-stable broadband MHz electromagnetic absorption, but also has strong practical application potential.

## Supplementary Information

Below is the link to the electronic supplementary material.

Supplementary file1 (DOCX 12400 KB)
