# Supplementary material for: Modulating Electromagnetic Genes Through Bi-Phase High-Entropy Engineering Toward Temperature-Stable Ultra-Broadband Megahertz Electromagnetic Wave Absorption
Source: Nanomicro Lett. 2025 Feb 25;17:164. doi: 10.1007/s40820-024-01638-4 (PMC11850694; doi:10.1007/s40820-024-01638-4)
Supplement: Supplementary file 1 — Supplementary file1 (DOCX 12400 KB) [file 40820_2024_1638_MOESM1_ESM.docx]

Supporting Information for

**Modulating Electromagnetic Genes through Bi-Phase High-Entropy Engineering toward Temperature-Stable Ultra-Broadband Megahertz Electromagnetic Wave Absorption**

Xiaoji Liu^1^, Yuping Duan^2^*, Nan Wu^3^, Guangming Li^4^, Yuan Guo^2^, Jiangyong Liu^2^, Ning Zhu^2^, Qiang Wang^1^, Lin Wang^1^, Zichen Xu^1^, Hao Wei^1^, Guojun Wang^1^, Zhijia Zhang^1^, Songsong Zhang^1^*, Wenjun Zhou^1^, Teng Ma^1^, Tongmin Wang^2^*

^1^ Qingdao Innovation and Development Base of Harbin Engineering University, Harbin Engineering University, Qingdao 266000, P. R. China

^2^ Key Laboratory of Solidification Control and Digital Preparation Technology, School of Materials Science and Engineering, Dalian University of Technology, Dalian 116085, P. R. China

^3^National Key Laboratory of Electromagnetic Effect and Security on Marine Equipment, China Ship Development and Design Center, Wuhan 430205, P. R. China

^4^ Wuhan Second Ship Design and Research Institute, Wuhan 430205, P. R. China

*Corresponding authors. E-mail: [duanyp@dlut.edu.cn](mailto:duanyp@dlut.edu.cn) (Yuping Duan); [zhangsongs@hrbeu.edu.cn](mailto:zhangsongs@hrbeu.edu.cn) (Songsong Zhang); [tmwang@dlut.edu.cn](mailto:tmwang@dlut.edu.cn) (Tongmin Wang)

**Supplementary Figures**

**Fig. S1** Composition analysis of selected areas for HEA, V300-2, A250-2, and A300-0.5

**Fig. S2** Composition analysis of selected areas for A300-1, A300-2, A300-6, and A350-2


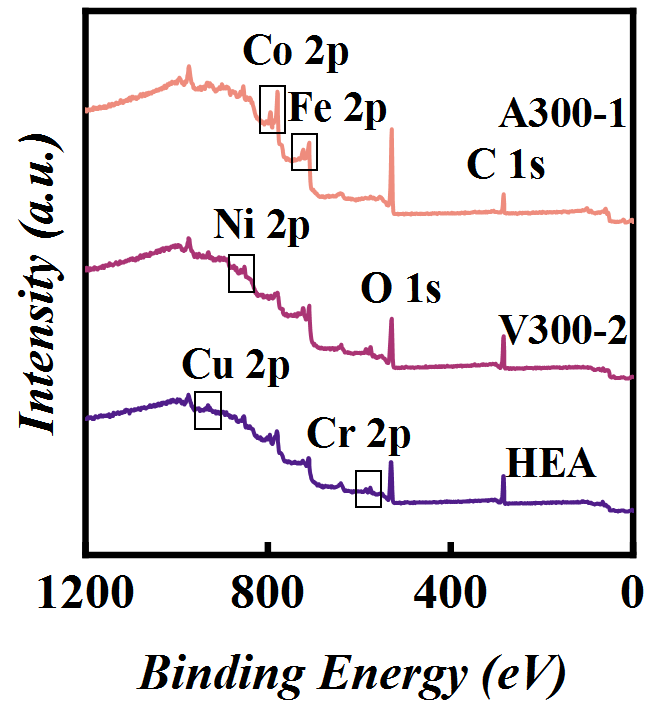


**Fig. S3** Broad scan XPS spectrum of HEA, V300-2 and A300-1

**Fig. S4** Fe 2p XPS spectra, Co 2p XPS spectra, Ni 2p XPS spectra, Cr 2p XPS spectra, and Cu 2p XPS spectra of HEA, V300-2 and A300-1

**Fig. S5** SEM image, and elements mappings of A350-2


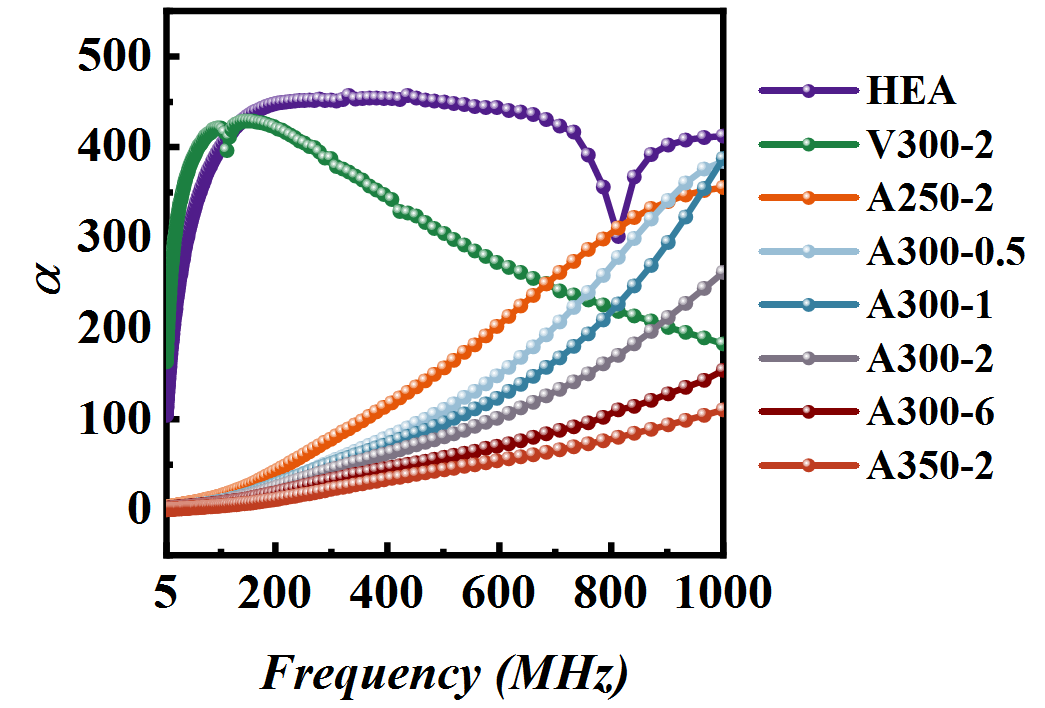


**Fig. S6** The attenuation coefficient *α* of HEA, V300-2, and HEA oxidized at different temperatures and times

**Fig. S7** Hysteresis loops of HEA and V300-2 versus temperature

**Fig. S8** Real permeability *μ′* and imaginary permeability *μ″* of HEA versus temperature

**Fig. S9** Magnetic loss tangent *tanδ_μ_* and dielectric loss tangent *tanδ_ε_* of A300-1 from −50 °C to 150 °C

**Fig. S10** The impedance matching *|Z_in_/Z_0_|* of A300-1 versus frequency and temperature

**Table S1** The particle size, purity, shape and source of the powders about the raw material powders

| Raw materials | Fe | Co | Ni | Cr | Cu |
| --- | --- | --- | --- | --- | --- |
| Particle size [μm] | 50 | 1–2 | < 50 | ≤ 50 | 20 |
| Purity | 99% | 99.5% | 99.5% | 99.5% | 99.8% |
| Shape | Spherical particles | | | | |
| Source of the powders | Shanghai Aladdin Bio-Chem Technology Co., LTD | | | | |

**Table S2** The saturation magnetization *M_s_*, coercivity *H_c_*, initial permeability *μ_i_* of HEA, V300-2, and HEA oxidized at different temperatures and times versus frequency

| Samples | *M_s_* [emu g^−1^] | *H_c_* [Oe] | *μ_i_* |
| --- | --- | --- | --- |
| HEA | 101.4 | 26.6 | 14.5 |
| V300-2 | 103.8 | 29.1 | 11.9 |
| A250-2 | 99.9 | 29.8 | 10.4 |
| A300-0.5 | 101.7 | 29.5 | 9.8 |
| A300-1 | 97.3 | 26.5 | 11.8 |
| A300-2 | 107.1 | 26.1 | 10.8 |
| A300-6 | 107.5 | 26.9 | 9.0 |
| A350-2 | 106.3 | 36.6 | 7.1 |

**Table S3** The performance comparison of representative MHz electromagnetic absorbers

| Samples | Thickness  [mm] | Frequency [MHz] | RL_min_  [dB] | EAB  [MHz] | Refs. |
| --- | --- | --- | --- | --- | --- |
| Co_2_O_3_-doped NiCuZn ferrite-0 wt% Co_2_O_3_ | 7.3 | 601 | −46 | 205 | [S1] |
| Co_2_O_3_-doped NiCuZn ferrite-0.5 wt% Co_2_O_3_ | 6.9 | 672 | −55 | 234 | [S1] |
| Co_2_O_3_-doped NiCuZn ferrite-1.0 wt% Co_2_O_3_ | 6.2 | 778 | −51 | 350 | [S1] |
| Co_2_O_3_-doped NiCuZn ferrite-1.5 wt% Co_2_O_3_ | 6.4 | 785 | −55 | 289 | [S1] |
| Ni_0.5_Co_0.5_Fe_2_O_4_/graphene | 4 | 840 | −31 | 420 | [S2] |
| Biochar with FeNi_3_ | 6 | 760 | −10 | 240 | [S3] |
| NiZn ferrite | 8 | 617 | −21 | 600 | [S4] |
| CIPs/ZnO/graphene | 4 | 480 | −46 | 410 | [S5] |
| FeCoNiCr_0.4_Cu_0.2_ HEAs/resin | 5 | 858 | −10 | 237 | [S6] |
|  | 6 | 760 | −13 | 278 |  |
|  | 7 | 685 | −16 | 284 |  |
|  | 8 | 617 | −21 | 270 |  |
| FeCoNiCr_0.4_Cu_0.2_ HEAs/SiO_2_ | 5 | 1000 | −8 | 82 | [S7] |
|  | 6 | 1000 | −10 | 281 |  |
|  | 7 | 1000 | −13 | 402 |  |
|  | 8 | 1000 | −15 | 488 |  |
| FeCoNiCr_0.4_Cu_0.2_ HEAs/CSFMs/SiO_2_ | 5 | 733 | −15 | 429 | [S8] |
|  | 6 | 596 | −18 | 361 |  |
|  | 7 | 519 | −22 | 328 |  |
|  | 8 | 452 | −27 | 265 |  |
| This work (−50 °C) | 5 | 638 | −13 | 593 | / |
| This work (−10 °C) | 5 | 596 | −13 | 620 | / |
| This work (30 °C) | 5 | 596 | −13 | 633 | / |
| This work (70 °C) | 5 | 556 | −12 | 646 | / |
| This work (110 °C) | 5 | 537 | −12 | 658 | / |
| This work (150 °C) | 5 | 484 | −11 | 691 | / |

**Supplementary References**

1. S. Yan, S. Liu, J. He, H. Luo, L. He et al., Effects of Co_2_O_3_ on electromagnetic properties of NiCuZn ferrites. J. Magn. Magn. Mater. **452**, 349–353 (2018). <https://doi.org/10.1016/j.jmmm.2017.12.108>
2. P. Yin, Y. Deng, L. Zhang, W. Wu, J. Wang et al., One-step hydrothermal synthesis and enhanced microwave absorption properties of Ni_0.5_Co_0.5_Fe_2_O_4_/graphene composites in low frequency band. Ceram. Int. **44**, 20896–20905 (2018). <https://doi.org/10.1016/j.ceramint.2018.08.096>
3. P. Yin, L. Zhang, Y. Wang, H. Rao, Y. Wang et al., Combination of pumpkin-derived biochar with nickel ferrite/FeNi_3_ toward low frequency electromagnetic absorption. J. Mater. Sci. Mater. Electron. **32**, 25698–25710 (2021). <https://doi.org/10.1007/s10854-020-04285-8>
4. L. He, L. Deng, Y. Li, H. Luo, J. He et al., Design of a multilayer composite absorber working in the P-band by NiZn ferrite and cross-shaped metamaterial. Appl. Phys. A **125**, 130 (2019). <https://doi.org/10.1007/s00339-019-2422-2>
5. P. Yin, L. Zhang, J. Wang, X. Feng, K. Wang et al., Low frequency microwave absorption property of CIPs/ZnO/Graphene ternary hybrid prepared *via* facile high-energy ball milling. Powder Technol. **356**, 325–334 (2019). <https://doi.org/10.1016/j.powtec.2019.08.033>
6. X. Liu, Y. Duan, Z. Li, H. Pang, L. Huang et al., FeCoNiCr_0.4_CuX high-entropy alloys with strong intergranular magnetic coupling for stable megahertz electromagnetic absorption in a wide temperature spectrum. ACS Appl. Mater. Interfaces **14**, 7012–7021 (2022). <https://doi.org/10.1021/acsami.1c22670>
7. X. Liu, Y. Duan, Y. Guo, H. Pang, Z. Li et al., Microstructure design of high-entropy alloys through a multistage mechanical alloying strategy for temperature-stable megahertz electromagnetic absorption. Nano-Micro Lett. **14**, 142 (2022). <https://doi.org/10.1007/s40820-022-00886-6>
8. X. Liu, Y. Duan, Y. Guo, Z. Li, J. Ma et al., *In situ* construction of complex spinel ferrimagnet in multi-elemental alloy for modulating natural resonance and highly efficient electromagnetic absorption. Chem. Eng. J. **462**, 142200 (2023). <https://doi.org/10.1016/j.cej.2023.142200>
